# Virus-Targeted Transcriptomic Analyses Implicate Ranaviral Interaction with Host Interferon Response in Frog Virus 3-Infected Frog Tissues

**DOI:** 10.3390/v13071325

**Published:** 2021-07-09

**Authors:** Yun Tian, Francisco De Jesús Andino, Collins N. Khwatenge, Jiuyi Li, Jacques Robert, Yongming Sang

**Affiliations:** 1 Department of Agricultural and Environmental Sciences, College of Agriculture, Tennessee State University, 3500 John A. Merritt Boulevard, Nashville, TN 37209, USA; ytian@tnstate.edu (Y.T.); ckhwaten@tnstate.edu (C.N.K.); jli4@tnstate.edu (J.L.); 2 Department of Microbiology and Immunology, University of Rochester Medical Center, Rochester, NY 14642, USA; francisco_dejesus@urmc.rochester.edu

**Keywords:** transcriptome, frog virus 3, Ranavirus, interferon signaling

## Abstract

Ranaviruses (*Iridoviridae*), including Frog Virus 3 (FV3), are large dsDNA viruses that cause devastating infections globally in amphibians, fish, and reptiles, and contribute to catastrophic amphibian declines. FV3’s large genome (~105 kb) contains at least 98 putative open reading frames (ORFs) as annotated in its reference genome. Previous studies have classified these coding genes into temporal classes as immediate early, delayed early, and late viral transcripts based on their sequential expression during FV3 infection. To establish a high-throughput characterization of ranaviral gene expression at the genome scale, we performed a whole transcriptomic analysis (RNA-Seq) using total RNA samples containing both viral and cellular transcripts from FV3-infected *Xenopus laevis* adult tissues using two FV3 strains, a wild type (FV3-WT) and an ORF64R-deleted recombinant (FV3-∆64R). In samples from the infected intestine, liver, spleen, lung, and especially kidney, an FV3-targeted transcriptomic analysis mapped reads spanning the full-genome coverage at ~10× depth on both positive and negative strands. By contrast, reads were only mapped to partial genomic regions in samples from the infected thymus, skin, and muscle. Extensive analyses validated the expression of almost all of the 98 annotated ORFs and profiled their differential expression in a tissue-, virus-, and temporal class-dependent manner. Further studies identified several putative ORFs that encode hypothetical proteins containing viral mimicking conserved domains found in host interferon (IFN) regulatory factors (IRFs) and IFN receptors. This study provides the first comprehensive genome-wide viral transcriptome profiling during infection and across multiple amphibian host tissues that will serve as an instrumental reference. Our findings imply that Ranaviruses like FV3 have acquired previously unknown molecular mimics, interfering with host IFN signaling during evolution.

## 1. Introduction

Frog virus 3 (FV3) is a large (~105 kb), double-strand DNA (dsDNA) virus belonging to the Ranaviruses genus (family *Iridoviridae)*, which comprises a group of emerging viruses that infect cold-blooded animals, including amphibians, fish, and reptiles [1,2]. FV3 infections were first reported in leopard frogs in the 1960s, and several virus isolates were obtained from cultured tissues/cells of both healthy frogs and tumor-bearing ones with renal carcinoma [1,2,3]. This implied tumorigenic potential; however, further studies demonstrated no etiological association of FV3 with the renal oncogenesis [1,2,3]. On the other hand, the association of FV3 with apparently healthy frogs indicates host-adaptive transmission and persistence, and may *de facto* cause diseases in other susceptible stages during the amphibian life cycle [4]. More studies have implicated Ranaviruses in the decline of amphibian populations worldwide [5,6,7,8]. FV3 represents the most frequently reported iridovirus for anurans. In North America, FV3 is widespread in wild amphibians, and is the only Ranavirus detected in turtles [6,9,10]. A recent study detected different FV3 lineages in wild amphibians in Canada, and these new FV3 isolates appears to have undergone genetic recombination with the common midwife toad virus (CMTV) [9]. CMTV represents another Ranavirus to affect various amphibians and reptile species and cause mortality events throughout Europe and Asia [9,10]. These findings reinforce the urgency to study ranaviral biology to face the bio-ecological threat from current catastrophic amphibian decline and negative impacts in aquaculture [1,2,3,4,5,6,7,8,9,10].

Among various Ranaviruses accounting for epizootics in amphibians, fish, and reptiles, FV3 is the best-characterized model and the prototype of the genus Ranavirus [1,2]. Historically, FV3 studies have provided insights into Ranavirus biology, including relevant characterization of highly methylated and phage-like genetic DNA, two-stage viral genome replication, temporal transcription, and virus-mediated arrest of the host response [2,11]. Prompted by early studies of FV3’s DNA synthesis occurring at the two-stage fashion between the nucleus and cytoplasm [11], more comparative genomic studies of large nuclear and cytoplasmic DNA viruses (NCLDVs) of eukaryotes have revealed the monophyletic origin of four viral families: *poxviruses, asfarviruses, iridoviruses,* and *phycodnaviruses* [12,13,14]. As recent proposals extend NCLDVs to include three other taxonomic families (*Ascoviridae*, *Marseilleviridae*, and *Mimiviridae*) and new founding members of other types of giant dsDNA viruses, advances in Ranavirus research contribute to delineate viral evolution and host tropism diversity among iridoviruses and NCLDVs, which can unveil evolutionary links among viruses associated with different cellular life forms [13,15]. However, in spite of the general characterization of FV3 replication and infection, the transcriptomic profile of the many viral genes and the precise roles of most viral proteins of FV3 and most other Ranaviruses remain elusive.

Early pioneering studies have resolved 47 viral RNAs and 35 viral proteins using gel electrophoresis in FV3-infected fish cells, and temporally classified them into early, immediate delay, and late genes along the viral infection cycle [16,17]. The first report of transcriptomic analysis used both microarray hybridization and RT-PCR validation to examine the expression of all 98 coding genes (or open reading frames, ORFs) as annotated in the FV3’s reference genome [18]. In that study, Majji et al. identified 33 immediate early (IE) genes, 22 delayed early (DE) genes, and 36 late (L) viral genes, while seven remaining genes were undetermined. As was postulated for the temporal class of FV3’s genes in general, early genes (including both IE and DE) encode putative regulatory factors, or proteins that act in nucleic acid metabolism and immune regulation, whereas products of L genes are involved in the virion packaging, assembly, and cellular interaction for viral release [18]. Notably, all of these previous FV3 gene transcription studies were performed in vitro using a fathead minnow (FHM) fish cell line model [16,17,18]. Thus, to date, FV3 transcriptomic profiling in vivo in infected host is lacking.

To complement recent virome studies and novel Ranavirus isolations, it is imperative to characterize *de novo* FV3’s transcriptome and conduct gene functional analysis using next generation sequencing (NGS)-facilitated metagenomics approaches [19,20]. To establish a procedure for unbiased analyses of ranaviral gene expression on a genome scale, we have performed a whole transcriptomic analysis (RNA-Seq) using total RNA samples containing both the viral and cellular transcripts from FV3-infected frog tissues. Two FV3 strains, a wild type (FV3-WT) and an ORF64R-knockout strain (FV3-∆64R), were used for comparison [21,22]. The gene 64R encodes a caspase-like activation and recruitment domain decoy (vCARD)-like molecule postulated to serve as an immune evasion gene. Recombinant FV3-∆64R virus exhibits attenuated virulence and growth in vivo, and exhibits a different host-pathogen interaction compared to wild-type FV3 [21,22]. In accordance with previous studies showing that FV3 replicates in multiple amphibian tissues [16,17], our virus-targeted transcriptomic analysis specifically mapped reads spanning the full-genome coverage at ~10× depth on both positive and negative strands in samples from the infected intestine, liver, spleen, lung, and especially kidney. In contrast, reads were only mapped to fragmental regions in samples from the infected thymus, skin, and muscle. Importantly, no viral transcript reads were detected in all control mock-infected tissue samples, indicating a well-controlled experimental handling and contamination-free processing. Our analyses identified the expression of most of the 98 annotated ORFs and profiled their differential expression in a tissue-, virus-, and temporal class-dependent manner. Furthermore, we used a reverse-genetic approach to functionally identify viral putative ORFs that encode hypothetical proteins, particularly those containing viral mimicking domains analogical to that in host interferon (IFN) regulatory factors (IRFs) or IFN receptors, especially for the type III IFNs. As a cardinal antiviral mechanism diversified along tetrapod evolution, the IFN system comprises three types of IFNs (type I, II, and III) that are classified mainly based on their molecular signatures and type-specific cognate receptors [23,24,25]. IFNs induce diverse immune responses extensively characterized in antiviral responses, and are involved in immunomodulatory processes through signaling cascades via respective IFN receptors and various IRFs [23,24,25,26]. Previous studies have determined the key position of amphibians in IFN evolution [24,25], and the alteration of viral infection in the IFN response in FV3-infected frogs [21,22]. The functional analyses may provide a mechanistic explanation about the viral interference of IFN responses in FV3-infected cells/tissues, and provide new insights into the evolutionary arms race between the Ranavirus and quickly evolving amphibian IFN system [21,22,23,24,25]. Our study thus provides the first virus-targeted, genome-wide transcriptome profiling of a large DNA virus during real amphibian host infection and uncovers the potential function of hypothetical proteins in the context of the virus-host interaction.

## 2. Materials and Methods

### 2.1. Animals and Virus

Outbred specific pathogen-free adult (1–2 years old) frogs were obtained from the *X. laevis* research resource for immunology at the University of Rochester [27]. All animals were handled under strict laboratory and University Committee on Animal Resources regulations (Protocol number: 100577/2003-151; approved by the Committee on Animal Resources Regulations at University of Rochester Medical Center on September 13, 2018, and expiration date September 13, 2021). Two FV3 strains, a wild-type (FV3-WT) and an ORF64R-desrupted strain (FV3-∆64R), were used for comparison. Frog virus 3 (FV3) stock preparation and animal infection were conducted as previously described [21,22]. In brief, fathead minnow (FHM) cells (ATCC^®^ CCL-42) or baby hamster kidney-21 (BHK-21) cells (ATCC^®^ CCL-10) were maintained in the suggested medium (DMEM or MEM; Invitrogen) supplemented with 10% fetal bovine serum (Invitrogen, Waltham, MA, USA), penicillin (100 U/mL), and streptomycin (100 μg/mL) at 30 °C with 5% CO_2_. FV3 was grown by a single passage in FMH or BHK-21 cells, and virus stocks were purified by ultracentrifugation on a 30% sucrose gradient. The virus load was assessed by plaque assays on a BHK-21 monolayer under an overlay of 1% methylcellulose (ATCC^®^ CCL-102). Virus stocks were titrated using plaque assays of serially diluted viral stocks on BHK-21 monolayers to express as plaque forming units (PFU), as previously described [21,22].

### 2.2. Animal Infection and Tissue Sampling

Adult frogs at comparable ages and body weights were randomly allotted into mock controls and infected groups (*n* = 5 per group). Animal infections were conducted by intraperitoneal (i.p.) injection of each animal with FV3-WT or FV3-∆64R at 1 × 10^6^ PFU in 100 μL of amphibian phosphate-buffered saline solution (APBS) or only APBS for mock controls. At 0, 1, 3, and 6 days post-infection, animals were euthanized by immersion in bicarbonate buffered 0.5% tricaine methane sulfonate (MS-222), and indicated tissues were sampled and pairwise allotted for classical viral titration and gene expression analyses. The samples of three days post-infection were selected for further unbiased or targeted transcriptomic studies, as diagramed in Figure S1 [21,22].

### 2.3. DNA/RNA Extraction and qPCR FV3 Gene Copy Assays

Total RNA and DNA were extracted from frog tissues and cells using a TRIzol reagent (Invitrogen) for PCR-based assays or a column-based RNA/DNA/protein purification kit (Norgen Biotek, Thorold, ON, Canada) for transcriptomic analysis. RNA integrity and concentration were evaluated with a NanoDrop 8000 spectrometer (NanoDrop, Wilmington, DE, USA) and an Agilent 2100 Bioanalyzer (Agilent Technologies, Santa Clara, CA, USA) to ensure RNA samples with A260/A280 > 1.8 and RNA integrity number (RIN) > 7.0 qualified for the construction of sequencing libraries [28,29].

Quantitative PCR (qPCR) analysis was performed using 150 ng/reaction of DNA templates in an ABI 7300 real-time PCR system and PerfeCta SYBR green FastMix, ROX (Quanta, Plain City, OH, USA). To measure the FV3 genome copy number based on detection of FV3gorf60R, which encodes a viral DNA polymerase II (Pol II), a qPCR was performed against a standard curve generated using a serially diluted template DNA containing 10^1^ to 10^10^ vDNA Pol II DNA copies cloned in a plasmid, as previously described [21,29].

### 2.4. Transcriptomic Assays (RNA-Seq)

RNA samples used for RNA-Seq sequencing library preparation were pooled from three qualified extractions of each group, as indicated above. For sequencing libraries construction, mRNA purification, fragmentation, construction of sequencing libraries, and sequencing were performed using the Illumina Pipeline (Novogene, Sacramento, CA, USA). Approximately 40 M clean reads per sample were generated for genome-wide transcriptomic analyses. The trimmed reads were further assembled and mapped to the reference genome/transcripts of *X. laevis* or FV3 virus through Xenbase [30] or NCBI genome ports [31], respectively. Only data for the virus-targeted transcriptome were reported here. The workflow of RNA-Seq analysis and data representative of general quality and comparability of the transcriptome data are shown in Supplemental Figure S1. Software used for reads mapping, quantification, differential analysis, sequential gene ontology (GO), and pathway analysis is listed in Supplemental Table S1. Significantly and differentially expressed genes (DEGs) between two treatments were determined using DeSeq and edgeR packages and visualized using bar charts (FPKM) or heatmaps (Log2 fold ratio), as previously described [29]. The transcriptomic dataset was deposited in the NIH Short Read Archive linked to a BioProject with an accession number of PRJNA705195.

### 2.5. Novel Viral Gene Prediction and Functional Analysis

We conducted extensive sequence- or pattern-based Blast searches against the FV3 reference genome (GenBank Accession No. NC_005946.1) using conservative domains in *Xenopus* proteins of IFN signaling, especially those of IFN receptors and IFN regulatory factors (IRFs). The Blast searching programs were mainly through the NCBI Blast portal [32] with the Expect threshold (E-value) adjusted to 1. Only viral proteins, which showed an E-value less than 0.5 and contain regions spanning almost full or full coverage of the functional domains in aligned host proteins, were selected for further simulation analyses. Further viral coding gene prediction was integrated to use both programs, fgenesV0 and fgenesV [33], and were annotated as novel open reading frames (Norf) if they were not annotated along the FV3 reference genome. The protein domain analysis was queried and extracted using the NCBI CDD database. The full-length sequences of the predicted hypothetical ORF/proteins are provided in the File S1. The GenBank accession numbers of all aligned gene/protein sequences are listed in Table 1 [24].

Multiple sequence alignments and views were done with a Jalview program. Protein structure models were simulated and visualized through combinative uses of the programs of PyMol, Chimera, and Phyre2 as described [29], as well as, primarily, a HDOCK server [34] for protein–protein or protein–DNA docking based on a hybrid algorithm of ab initio free docking. Without further indication in the legends, all programs were used under a defaulted condition.

### 2.6. Statistical Analysis

Statistical analysis was conducted using one-way analysis of variance (ANOVA) and Tukey’s post hoc test. A two-sample F test was used for significant evaluation between samples/treatments. A probability level of *p* < 0.05 was considered significant [24,28].

## 3. Results and Discussions

### 3.1. FV3 Infection and Comparative Viral Determination Between FV3-WT and FV3-∆64R Strains in the Kidneys of Adult Frogs

**Figure 1:**
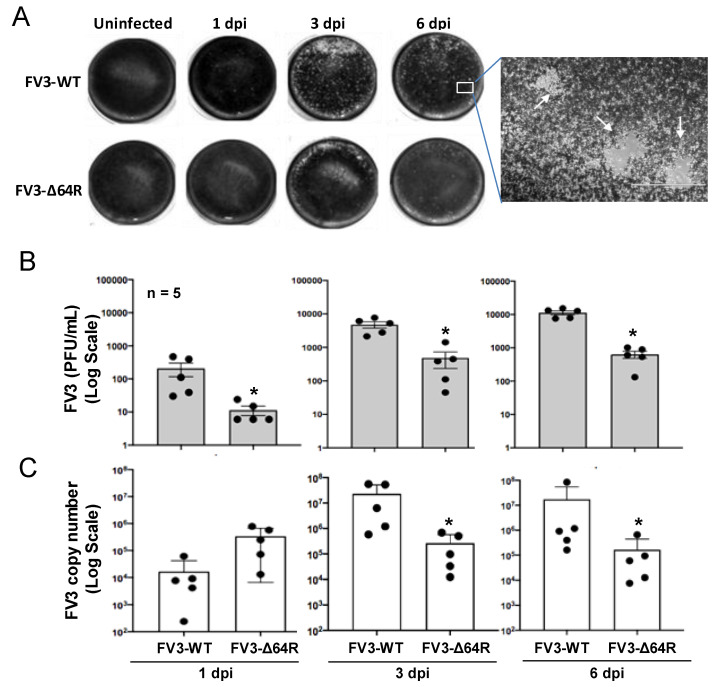
Viral plaque assays and genome copy number detection by quantitative PCR (QPCR). (**A**) For plaque assays, 1 mL of the virus-containing supernatant of tissue homogenate from an individual kidney sample was used to inoculate A6 cells (ATCC^®^ CCL-102™). FV3 plaques were counted at 1, 3, and 6 days post-infection (dpi) and imaged for representative wells. (**B**) Virus titers were calculated to present as PFU/mL average for each group/treatment. (**C**) Virus genome copies were examined in the DNA samples from the infected kidneys using a routine QPCR procedure to determine the FV3gorf60R gene copies in 150 ng DNA per reaction as described. * *p* < 0.05, *n* = 5 for (**B**,**C**).

FV3 infects anuran amphibians at various developmental stages, and is highly lethal in tadpoles. Adult frogs, by contrast, are more resistant to viral infection, and after viral clearance, a low level of quiescent viruses were isolated from apparently healthy frogs [21,22,28]. This indicated that adult frogs are more adaptive to the deathliness caused by the virus [22], and meanwhile serve as active carriers or reservoirs for the virus transmission, providing a valuable model for studying the virus–host coevolution [26]. In this context, we infected in laboratory-controlled conditions 1–2-year-old *X. laevis* frogs for well-controlled sample collection. As shown in Figure S1 and Figure 1, randomly allotted frogs were infected with either a FV3-WT or FV3-∆64R strain. Time points were chosen based on a previously published study to include an early innate immune response (1 dpi), intermediate response (3 dpi), and the peak of the adaptive T cell immune response (6 dpi) [3,4]. Both FV3 strains caused early productive infections, as shown with successful viral isolation from the kidney tissue homogenates of infected frogs (Figure 1A). As anticipated, FV3-WT was more efficient in producing infectious virions compared to the FV3-∆64R mutant virus (with disruption of the ORF64R gene, encoding a putative interleukin-1 beta convertase and containing a caspase activation and recruitment domain (vCARD)), showing a 100–1000 magnitude difference compared to using a logarithmic scale of PFU at 1–6 dpi (Figure 1B). Similar infection patterns were also observed by measuring the virus genome copy number based on quantitative detection of the viral Orf60R gene, which encodes a virus DNA polymerase II unit (Pol II) (Figure 1C). However, the viral genome copy number of FV3-WT kept increasing through 1–6 dpi, whereas FV3-∆64R genome copies reached a higher level than the wild type at 1 dpi and kept a similar level through the tested period without much increase (Figure 1C). From daily observations throughout the infection process, infected frogs behaved similarly to the mock-infected controls, and no outliers per clinical observation or virus diagnosis were identified, which were averagely qualified for sample collection, as designed for further transcriptomic processing (Figure S1).

### 3.2. Virus-Targeting Transcriptome Analysis and Difference Dependent on Tissue Types and FV3 Strains

**Figure 2:**
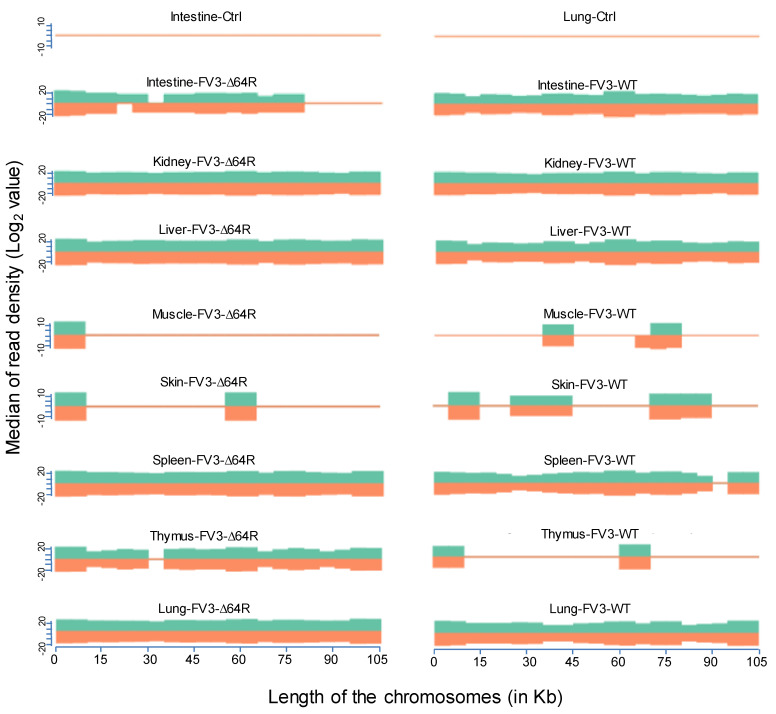
Virus-targeted transcriptome analysis in the control and infected samples at 3 dpi. Shown is the distribution plots of mapped reads in the FV3 genome (GenBank accession no. NC_005946.1). The x-axis shows the length of the genome (in Kb, 105 Kb of FV3), and the y-axis indicates the log2 of the median of the read density. Green and red indicate the positive and negative strands, respectively. Note, no FV3 transcript reads were obtained from the control (Ctrl) non-infected samples (shown only from the intestine and lung).

FV3’s genome encodes 98 putative coding genes (FV3gorf1-98) as annotated along its reference genome [18]. Previous microarray analysis plus RT-PCR validation determined the expression of all 98 FV3 ORFs, indicating full-genome transcribing capacity during FV3 infection in the FHM cell line [18]. Consistent with the microarray analysis, our comprehensive unbiased transcriptome analysis based on de novo deep sequencing revealed FV3 gene-specific reads spanning the full FV3 genome at ~10× depth in samples from the infected intestine (FV3-WT only), liver, spleen (FV3-∆64R only), lung, and particularly kidney (Figure 2). In addition, partial genome coverage or regional detection were obtained in FV3-infected muscle, skin, and thymus tissues (Figure 2). Importantly, no FV3-specific reads were detected from any sham-infected control tissues, ruling out cross-contamination and validating our sample handling procedures.

**Figure 3:**
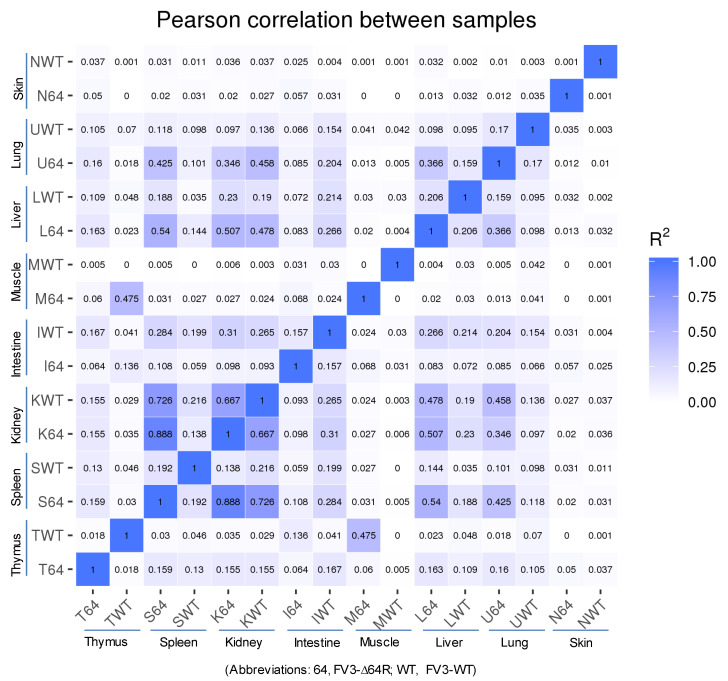
RNA-Seq correlation among the samples. Heat maps of the correlation coefficient between samples are shown. Numbers indicate the square of the Pearson coefficient (R2). The closer the correlation coefficient was to 1, the greater the similarity of the samples. Generally low R2 values indicated a dramatic difference between different tissues and two FV3 strains.

Statistical analyses of the RNA-Seq results revealed several interesting aspects: (1) FV3 may maintain a more complex transcript mixture in vivo than in a uniform cell line related to unsynchronized infection stages upon diverse cell types in tissues. Given that the viral transcripts were significantly detected in the kidney, spleen, liver, lung, thymus, and intestine, we concluded that the differential viral gene expression in different tissues resulted primarily from a systemic virus–host interaction initiated from intraperitoneal injection. (2) FV3-specific reads were significantly enriched in an increasing order in the intestine, lung, liver, spleen, and predominantly in the kidneys, but much less abundant in the skin and muscle, where FV3 replication may be negligible. (3) The transcript profiles of the FV3-∆64R mutant were nearly identical to the FV3-WT in the kidney, but qualitatively and quantitatively very different in other tissues. (4) The unbiased transcriptome study detected transcripts of the FV3 genome almost equivalently along both positive and negative strands, which confirmed the existence of viral coding genes at both strand orientations (Figure 2). Further co-expression Venn analysis confirmed a virus strain (FV3)-dependent difference of gene transcription among most tested tissues, as both WT and FV3-∆64R shared near identical transcript profiles of all 98 annotated FV3gorfs in the kidney. Interestingly, although WT FV3 genes were more efficiently transcribed than FV3-∆64R in the intestine, skin, and kidney, the recombinant mutant virus actually had much more transcripts in the thymus, liver, lung, and particularly spleen. This implies that the disruption of the FV3gorf64R gene, which encodes a putative interleukin-1 beta convertase containing caspase recruitment domain (vCARD), may change viral transcription dynamics and the tissue/cell tropism of FV3 infection in amphibians (Figure 2). As shown in Figure 3, the Pearson correlation analysis demonstrated a generally low cross-sample correlation, except between the FV3-∆64R-infected spleen and kidney, further indicating that both tissue types had a top priority to support FV3 infection and full-scale gene expression (Figure 3).

### 3.3. Genome-Wide Differential Expression Analysis of FV3 Coding Genes

**Figure 4:**
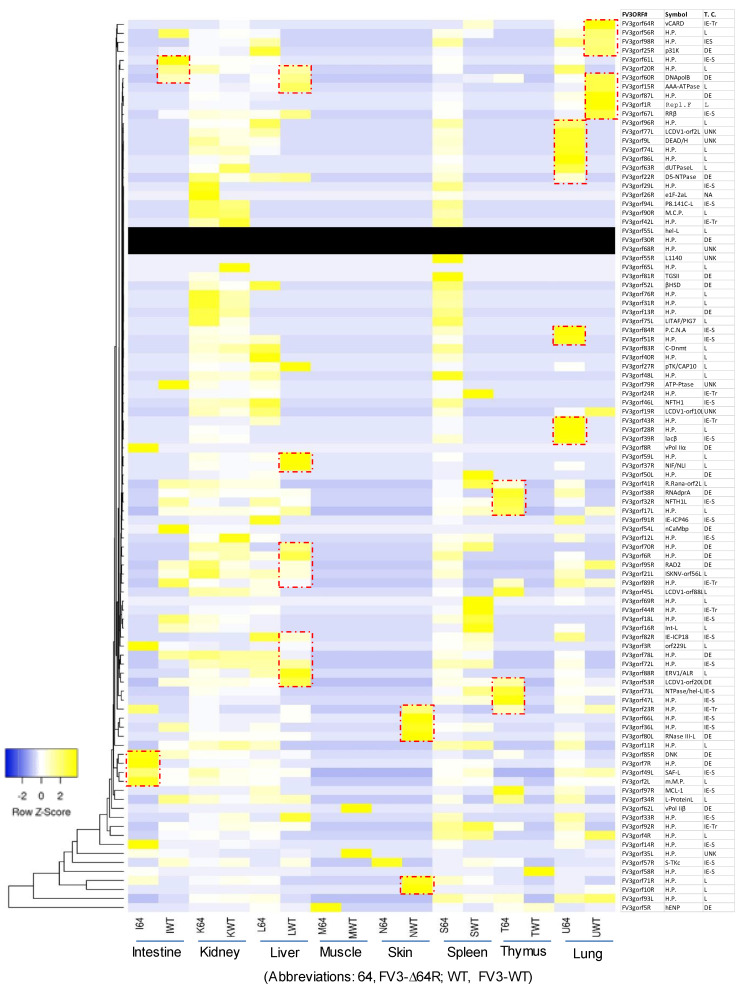
Heatmap and cluster analysis of differential expression of all validated/putative ORFs for coding genes along the FV3 genome. FPKM values were used as for paired-end RNA-Seq per differential gene expression and cluster analysis, clustered using the log_10_(FPKM + 1) values. Yellow denotes genes with high expression levels, and blue denotes genes with low expression levels. The color range from yellow to blue represents the log_10_(FPKM + 1) value from large to small. The ORFs sit in the same or close clusters that have similar expression patterns across the samples. The black rows indicate that the expression of the corresponding ORFs has not been detected, implying a silent or nonproductive transcription. The table at the right lists the 98 annotated open reading frames (ORFs) in the FV3 genome and the corresponding designation of gene symbols as defined under the GenBank FV3 reference genome (NC_005946.1). The red line frames indicate higher expressed gene clusters in the infected tissues other than the kidney and spleen, where FV3 primarily showed high expression in general. Other abbreviations: FPKM, fragments per kilobase of transcript per million mapped reads; H.P., hypothetical proteins; T.C., temporal class.

Figure 4 presents a heat map and cluster analysis of differential expression based on all 98 putative ORFs of the annotated FV3 genome. Using the FPKM (fragments per kilobase of transcript per million mapped reads) values as standardization for paired-end RNA-Seq analysis, the results showed a tissue-dependent expression of various viral genes across the genome-wide ORF panel. Again, most FV3 genes were highly expressed in the kidney and spleen, but very few genes also showed high expression levels in other tissues, including the intestine, liver, thymus, and lung (red line framed in Figure 4). Within major gene clusters with similar tissue expression patterns, the correlation to their functional relevance or temporal class was observed to some extent. However, this should not be considered as a general reference for interpreting viral gene function within each cluster because, even when genes shared cross-tissue expression patterns, they apparently had different putative functions, and were ascribed to different temporal classes (Figure 4). Again, most viral genes had differential expression patterns between the FV3-WT and FV3-∆64R strains, suggesting that the putative cCARD gene plays an important role in mediating the virus–host interaction, which influences viral transcription. Among the 98 annotated FV3gorfs, no specific reads were mapped to three viral genes of FV3gorf-55L, -30R, and -68R (black rows in Figure 4), which implies that, during infection in vivo, these ORFs were not transcribed or underwent transcript decay in the tested tissues. However, this does not exclude their potential expression in other tissues/cell types and/or other host species, since they have been previously detected in the FHM cell culture by microarray [18]. This underscores the need of extensive comparative analysis of the virus transcriptome at different time points post-viral infection in both tadpoles and adult frogs of different species using the established NGS platform (Figure S1 and Table S1).

### 3.4. Differential Expression Analyses According to the Temporal Class of FV3 Coding Genes

**Figure 5:**
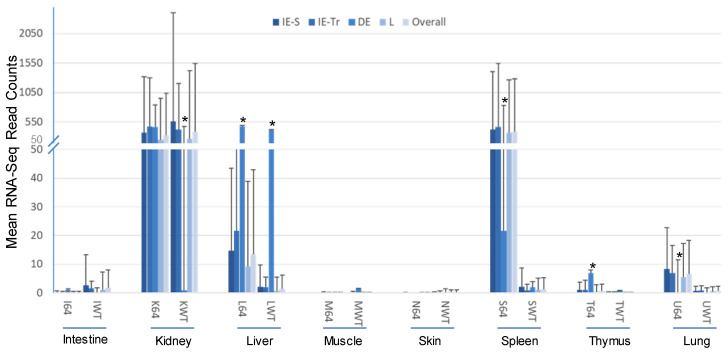
Differential expression of FV3 ORF coding genes based on their temporal classification in the viral infection. FV3 gene expression is temporally regulated in a coordinated fashion, leading to the sequential appearance of immediate early (IE), delayed early (DE), and late (L) viral transcripts. The IE genes include immediate early stable messages (IE-S) and immediate early transient messages (IE-Tr). Transcriptomic analysis of the expression of the 98 total annotated FV3 ORF coding genes (*Xenbase*) indicates a differential expression pattern dependent on the gene class, virus strain, and especially tissue types. * *p* < 0.05, *n* > 10, compared to the overall average. Other abbreviations: 64, FV3-∆64R; WT, FV3-WT; Overall, genes in all of the classes.

As mentioned, it is widely assumed that early (E) genes include encoding regulatory factors or proteins that mediate nucleic acid metabolism and immune interaction, whereas late (L) genes primarily take part in DNA packaging and virion assembly and releasing. Transcripts of FV3’s E genes are further classified into immediate early stable messages (IE-S), immediate early transient messages (IE-Tr), and delayed early (DE) transcripts [16,17,18]. Figure 5 categorizes the differential expression of FV3 ORF coding genes based on their temporal classification, as determined above. Similar tissue- and virus strain-dependent expression patterns were clearly demonstrated with a 100–1000 fold higher viral transcription of most temporal classes in the kidney (both strains), spleen (FV3-∆64R only), and DE in the liver (FV3-∆64R only). The temporal class exhibiting the most differential expression pattern was the DE genes. Notably, the DE transcripts were: (a) dramatically different in kidneys between FV3-∆64R (comparably as high as the overall average) and FV3-WT (significantly lower than the overall average); (b) significantly higher on average than the other temporal classes in the infected livers; and (c) significantly lower than other temporal classes in the FV3-∆64R-infected spleen and lung, but higher in the thymus (Figure 5). These observations indicate that FV3-WT has an optimal tissue-specific regulation (or inter-tissue collaboration) of DE transcription between the kidney and livers, whereas FV3-∆64R seems deficient in this capacity, suggesting that the FV3gorf64R gene plays a critical role in the regulation of DE gene expression in FV3-infected frogs.

**Figure 6:**
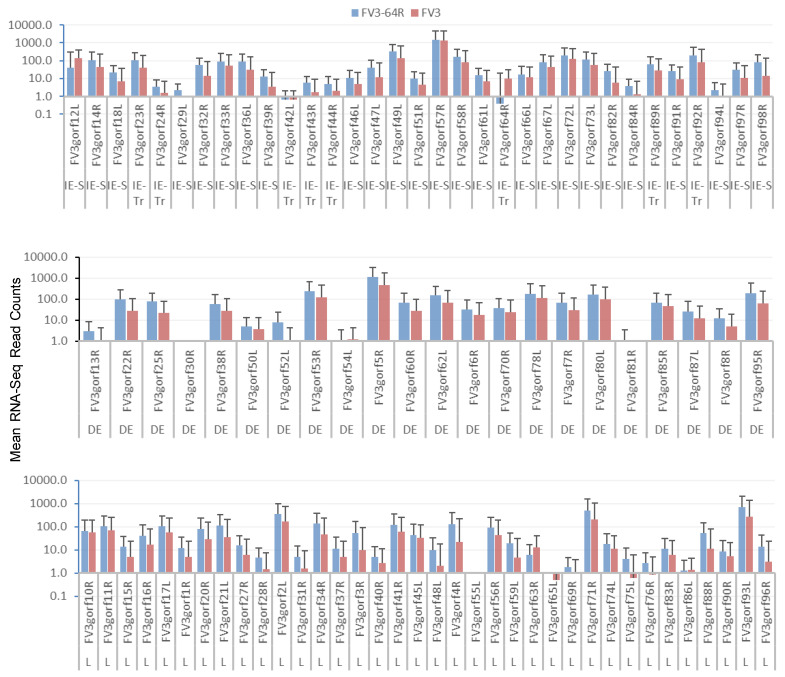
Differential expression of individual genes of FV3 between the FV3-∆64R and FV3-WT infected groups. The genes are categorized based on their sequential classes as immediate early (IE), delayed early (DE), and late (L) viral transcripts. RNA-Seq reads are presented as averages across the eight types of different tissues (i.e., intestine, kidney, liver, muscle, skin, spleen, thymus, and lung) to demonstrate that the differential expression is dependent on the gene and virus strain. Note that the y-axis is in a log_10_ scale. Other abbreviations: 64, FV3-∆64R; IE-S, immediate early stable messages; IE-Tr, immediate early transient messages; WT, FV3-WT.

**Figure 7:**
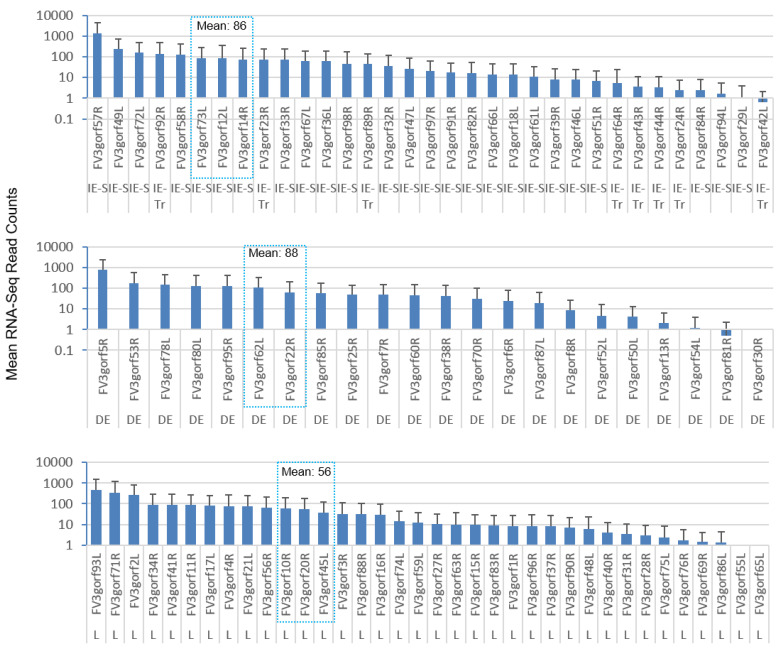
Sorted expression levels of individual FV3 ORF coding genes to show the relative expression order across and within each temporal gene classes. The genes are categorized based on their sequential classes as immediate early (IE), delayed early (DE), and late (L) viral transcripts. RNA-Seq reads are presented as averages across all samples to demonstrate the differential expression based on each gene in general at three days post-infection. Note that the y-axis is in a log10 scale, and is the difference of the mean values between each temporal gene classes. The genes, which have an expression level close to the group means (framed by the blue dashed line), should serve as better gene markers for the estimation of viral genome copies for classical QPCR detection. Other abbreviations: 64, FV3-∆64R; IE-S, immediate early stable messages; IE-Tr, immediate early transient messages; WT, FV3-WT.

Figure 6 shows the differential expression profiles of individual genes between FV3-∆64R and FV3-WT infected groups. Although many genes had comparable expression levels between the FV3-∆64R and FV3-WT, the average transcript counts of the WT strain were lower, particularly of the DE and L classes. Because mutant virus-infected tissues contained less infectious virions (Figure S1), it is plausible that FV3-∆64R underwent less efficient virus packaging and resulted in higher accumulation of gene transcripts [18,22]. In Figure 7, we present the averaged expression levels of all annotated FV3 genes sorted by decreasing order across and within each temporal gene class. Data show that the E gene group had a two-fold higher group-wide median value than the L genes. It is noteworthy that the genes with an expression level close to the group means (framed by the blue dashed line) are likely to serve as better gene markers for the estimation of viral genome copies by classical QPCR [21,22,28].

### 3.5. Detection of Interferon Regulatory Factor Domain or Fibronectin Type 3 Domain in Several FV3 Hypothetical Proteins

Several FV3 genes were previously assessed using FV3 knockout mutants, including FV3−Δ64R, FV3−Δ52L, and FV3−Δ82R, which contain molecular defects for regarding genes to putatively encode a vCARD-like protein, a β-hydroxysteroid dehydrogenase homolog (βHSD), and an IE-18kDa protein (ICP18), respectively [21,22]. Comparative examination of viral loads and responsive cytokine gene expression in macrophages or kidney tissues from frogs infected between these FV3 mutants and FV3-WT demonstrated reduced viral loads and an altered IFN and tumor necrosis factor (TNF)-response that were different in infected tadpole and adult frog tissues [21,22]. Based on functional aspects of their host homologs, both viral CARD-like and βHSD mimics were probably interfering with the regulation of host immune responses, particularly the inflammatory response [35,36]. Thus, these viral genes may indirectly intersect to the antiviral IFN response through a newly elucidated non-canonical epigenetic regulation [37,38]. Similarly, the FV3 ICP18 gene containing a DUF2888 domain shared within the Ranavirus clade appears to affect IFN signaling by unknown mechanisms. Therefore, we sought to determine potential ranaviral factors that may directly affect the virus–host interaction based on transcriptomic analysis. Through integrative uses of protein domain searching, amino acid sequence similarity, and structural analyses of hypothetical proteins encoded in the FV3 genome, we identified eight FV3 hypothetical proteins containing regions analogous to the interferon regulatory factor (IRF) domain or Fibronectin type 3 (FN3) domain (Table 1). As FN3 is a functional domain in cellular receptors conferring the recognition of IFNs and other cytokines, IRF domains of IRF transcription factors are characterized by the DNA-binding capacity in the promoters of various IFN-stimulated genes (ISGs), including IFNs themselves [39,40,41]. Because of the critical role of IFN receptors and IRFs in IFN-mediated antiviral immunity, various antagonisms have been identified [39,40,41]. Particularly in the NCLDV group that has a large DNA genome, viral mimics counteracting IFN receptors and IRFs have been studied (e.g., human herpesviruses [41,42,43,44]). However, no similar ranaviral mimics have been elucidated so far, even though various antagonistic effects on the IFN response by FV3 infection have been observed [21,22,28].

**Figure 8:**
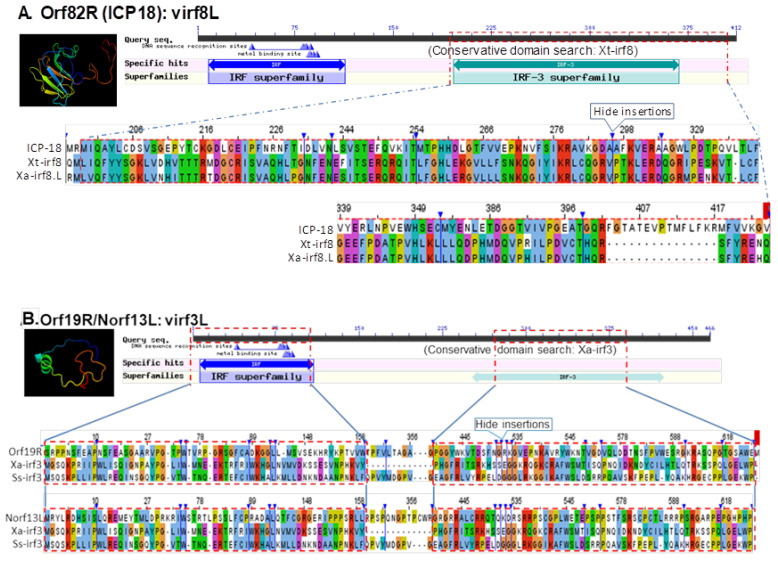
Identification of putative interferon regulatory factor domain (IRF) in FV3 hypothetical proteins, which are potentially virus-coding molecular mimics able to interfere with the host antiviral interferon signaling. (**A**) Orf82R refers to FV3orf82R spanning 89,450–89,923 nt region on the positive strand of the FV3 genome (NC_005946.1), and encodes a hypothetical protein ICP-18 of 157 AA. It contains the second IRF-like domains at ~30% AA sequence similarity to the second IRF domain detected at *Xenopus* IRF8 proteins, as illustrated. (**B**) Orf19R refers to FV3orf19R spanning the 21,916–24,471 nt region on the positive strand of the FV3 genome and encodes a hypothetical protein of 851 AA. It contains two IRF domains (partially second one) at its C-terminal ~500 AA. Norf13L ascribes a novel open reading frame (Norf) predicted using the FGEESV0 program, which spans the 14,685–15,092 nt region of the negative strand of the FV3 genome and encodes a hypothetical protein of 135 AA. It contains two nearly consecutive IRF domains (partially a second one) as aligned to irf3 homologs. The full-length sequences of the predicted hypothetical ORF/proteins are provided in File S1. The collective information of other IRF-like domain-containing proteins and GenBank accession numbers of the aligned protein sequences are listed in Table 1. Putative protein domains were queried and extracted using the NCBI CDD database, and their tertiary structure was modeled using Phyre2 and PyMol programs. Multiple sequence alignments were obtained using a Jalview program.

As shown in Table 1, most of these viral mimics contain regions resembling one or two IRF domains (vIRFs) of various vertebrate IRF proteins, and two share similarity with the FN3 domains (vFN3) of IFN receptor subunits, forming the type III IFNs, i.e., IFN-λ receptor 1 (ifnlr1) and interleukin-10 receptor beta (il10rb). In Figure 8 and Figure S3, we performed amino acid similarity alignments of the identified vIRFs with respective IRF domains conserved among vertebrate IRF protein targets. The general IRF consensus comprises an N-terminal DNA-binding domain (DBD) with five typical tryptophan repeats (5W) and a C-terminal activation region (AD) containing an IRF-associated domain (IAD). As DBD is essential for the recognition of DNA motifs within conserved *cis*-regulatory elements (CRE) in the promoter region of IFN or ISG genes, more variable IAD mediates protein–protein interactions with other transcription factors, and hence defines the functional diversity of different members in the IRF family [40,41]. Comparable to vIRFs identified in human herpesviruses, FV3’s vIRFs share a high similarity of positive charged residues and an average ~30% amino acid sequence identity with DBD and/or AD domains in corresponding amphibian IRFs, but less similarity with the 5W pattern associated to the DBD domains [40,41,42,43,44,45]. The identified putative vIRFs exhibit a broad target potential on *Xenopus* IRF1/IRF2 (Norf76R), IRF3 (Norf13L and Orf19R), IRF4 (Orf41R), IRF6 (Norf42L), and IRF8 (Orf27R and Orf82R). In addition to the coding genes of the 98 FV3gorfs (Orf) annotated along the FV3 reference genome, some vIRFs were encoded by alternative coding frames (Norfs) and are newly predicted by this study with supportive transcriptomic data. This indicates an extended coding capacity of the FV3 genome beyond our previous understanding (File S1). Notably, the temporal class of these viral mimics (except Norfs) has been reported as unknown (UNK) or L in previous studies [16,17,18], except Orf82R (encoding ICP18) that is an IE gene [18,22]. This substantiates our previous observation about the increased stimulation of type I and III IFNs in FV3-∆18K infected tadpole and frog tissues [21,22]. Studies of human and murine IRFs have shown that IRF4 and IRF8 are highly expressed in lymphoid and myeloid immune cells, and are critical for B lymphocyte development and Th cell differentiation. Notably, IRF8 is required for IFN production by dendritic cells (DCs), particularly plasmacytoid DCs (pDCs) that are important IFN producers for early antiviral regulation [24,44,45]. Therefore, ICP18 in Ranaviruses is postulated to affect early antiviral IFN responses by targeting amphibian IRF8-mediated IFN responses in lymphocytes, including DCs and macrophages [22,45].

The main characterized roles of other IRFs include: (1) IRF1 and IRF2 regulate T cell activation and enhance the Th1 immune response; (2) IRF3 and IRF7 are engaged in IFN production, signaling downstream innate immune recognition of various intracellular pathogens, including viruses; (3) IRF5 is involved in the regulation of inflammation and apoptosis, is structurally similar to IRF6, and the regulates proliferation and differentiation of keratinocytes; and (4) IRF9 is part of the IFN-stimulated gene factor 3 (ISGF3) complex that transmits type I and III IFN signals [40,44,45]. The identification of vIRFs broadly targeting amphibian IRF1/2, IRF3, IRF4, IRF6, and IRF8 is supported by previous and current observations about FV3’s interaction with IFN-mediated immune responses in amphibians, which also indicates a general cross-species conservation, molecularly and functionally, of these amphibian IRFs in immune regulation (Table 1, Figure 8) [21,22,28,44].

**Figure 9:**
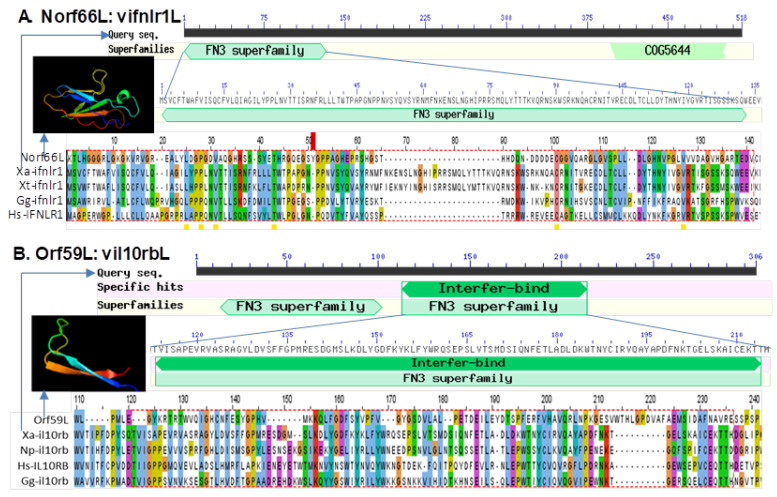
Detection of fibronectin type 3 domain (FN3) conserved in the IFN receptors of several FV3 hypothetical proteins, which are potential virus-coding molecular mimics able to interfere with the host antiviral interferon signaling. (**A**) Norf66L ascribes a novel open reading frame (Norf), which spans the 59,162–60,037 nt region of the negative strand of the FV3 reference genome and encodes a hypothetical protein of 291 AA. It contains an FN3 domain (residue 121–230 AA) similar to vertebrate interferon lambda receptor 1 (ifnlr1) isoforms regarding the sequence similarity and β-sheet containing structure. (**B**) Orf59L refers to FV3orf59L spanning the 65,956–67,014 nt region on the negative strand of the FV3 genome and encodes a hypothetical protein of 352 AA. It contains an FN3 domain region at 108–203 AA and is molecularly similar to that of *Xenopus* IL-10 receptor beta unit (il10rb). The full-length sequences of the predicted hypothetical ORF/proteins are provided in File S1. The collective information of other IFN-interfering, domain-containing proteins and GenBank accession numbers of the aligned protein sequences are listed in Table 1. The protein domain analyses were queried and extracted using the NCBI CDD database, the tertiary structures were simulated using Phyre2 and PyMol programs, and sequence alignments and view were simulated with a Jalview program.

Figure 9 shows the alignments of two vFN3 mimics that contain a relevant domain similar to vertebrate interferon lambda receptor 1 (ifnlr1) or IL-10 receptor beta unit (il10rb), which form a functional IFN receptor interacting with type III IFNs in responsive cells [39,40,41,42]. As illustrated in this study, Norf66L encodes a novel open reading frame (Norf), which spans a 59,162–60,037 nt region on the negative strand of the FV3 reference genome and encodes a hypothetical protein at 291 AA. It contains an FN3-like domain (residue 121–230 AA) similar to the ifnlr1 isoforms both in term of the sequence similarity and modeled β-sheet containing structure. By contrast, Orf59L refers to FV3orf59L spanning a 65,956–67,014 nt region on the negative strand of the FV3 genome and encodes a hypothetical protein at 352 AA. It contains an FN3 domain region at 108–203 AA and is molecularly similar to that of *Xenopus* il10rb. The detection of vFN3 mimics that primarily target type III IFN receptors is consistent with previous observations about FV3’s suppressive effect on IFN-λ expression in *Xenopus* [22,28], which generally concurs with our host-specific transcriptome analysis [46]. It is therefore likely that FV3 has strengthened its IFN antagonism during evolution to overcome the epithelial specific type III IFNs signaling pathway, particularly in tadpoles [22,28,47,48,49].

## 4. Conclusive Highlights

Frog virus 3 (FV3) represents a well-characterized model to study Ranavirus pathogens that are prevalent in worldwide habitats of amphibians, fish, and reptiles, and significantly contribute to the catastrophic amphibian decline [5,6,7,8,9,10]. Based on conventional and novel assignation of FV3 coding genes per their temporal expression fashion along the virus infection stages [16,17,18], the current study used an unbiased transcriptomic RNA-Seq analysis to profile and compare viral transcripts in various tissues of frogs infected with either FV3-WT or a FV3-∆64R strain defective for a gene encoding a CARD motive. The results revealed a full-genome coverage transcriptome annotated to almost all coding genes at ~10× depth on both positive and negative strands in RNA samples from the infected intestine, liver, spleen, lung, and especially kidney. In contrast, partial transcript coverage was detected in infected thymus, skin, and muscle tissue, suggesting inefficient viral replication in these tissues. Extensive analyses indicated a multi-organ infection pattern of FV3 infection in frogs and validated the in vivo expression of most annotated 98 ORFs, as well as their differential expression in a tissue-, virus strain-, and temporal class-dependent manner. About half of FV3’s coding genes have not yet been functionally determined in the scenario of the virus–host interaction. Our transcriptome-initiated functional analyses focused on putative ORFs that encode hypothetical proteins containing viral mimicking domains, such as host interferon (IFN) regulatory factors (IRFs) and IFN receptors. Our findings suggest that Ranaviruses like FV3 have acquired during evolution previously unknown molecular mimics interfering with host IFN signaling, which thus provide a mechanistic understanding about Ranavirus persistence in adult frogs. In summary, this study provides a comprehensive virus-targeted transcriptome analysis to profile the genome-wide gene expression of a large double-strand DNA virus, and uncovers the potential IFN-interfering function obtained by some ranaviral hypothetical proteins to perturb the virus–host interaction.

## Figures and Tables

**Table 1:** Viral genes that encode hypothetical proteins containing conserved domains and are potentially able to interfere with host interferon signaling.

Location on FV3 Genome	Viral Gene (Temporal) Designation	ORF/Protein Size	Validation/Prediction Algorithm	Analogy to IFN-Interfering Domain (E < 0.5 and ~30% id.)
14,685–15,092 (−)	Norf13L (UNK)	408 nt/135 aa	FGEESV0 (Markov chain-based)	IRF domains in Xa-irf3 (NP_001079588) Ss-irf3 (NP_001165753)
21,916–24,471 (+)	Orf19R (UNK)	2556 nt/851 aa	NCBI Annotation/FGEESV0	IRF domains in Xa-irf3 (NP_001079588) Ss-irf3 (NP_001165753)
33,728–36,640 (+)	Orf27R (L)	2913 nt/970 aa	NCBI Annotation/FGEESV0	IRF domains in Dr-irf8 (NP_001002622) Xt-irf8 (XP_004913664)
38,635–39,102 (−)	Norf42L (UNK)	468 nt/155 aa	FGEESV0	IRF domains in Dr-irf6 (NP_956892) Xt-irf6 (NP_001025493)
46,691–50,188 (+)	Orf41R (L)	3498 nt/1165 aa	NCBI Annotation/FGEESV0	IRF domain in Dr-irf4a (NP_001116182) Xt-irf4 (XP_002936464)
59,162–60,037 (−)	Norf66L (UNK)	876 nt/291 aa	FGEESV0	FN3 domain in Xa-ifnlr1 (XP_018097809) Xt-ifnlr1 (ACV32138)
65,956–67,014 (−)	Orf59L (L)	1059 nt/352 aa	NCBI Annotation/FGEESV0	FN3 domain in Xa-il10rb (XP_018101420) Hs-il10rb (NP_000619)

## Data Availability

All data from this study are available within this manuscript and its Supplementary Material.

## Supplementary Materials

The following are available online at https://www.mdpi.com/article/10.3390/v13071325/s1, Figure S1: The workflow and data showing general quality and comparability of the RNA-Seq transcriptome data. Table S1: Software List for data bioinformatic analysis. File S1: The full-length sequences of the predicted hypothetical ORF/proteins.
