# Supplementary material for: Virus-Targeted Transcriptomic Analyses Implicate Ranaviral Interaction with Host Interferon Response in Frog Virus 3-Infected Frog Tissues
Source: Viruses. 2021 Jul 9;13(7):1325. doi: 10.3390/v13071325 (PMC8309979; doi:10.3390/v13071325)
Supplement: Supplementary file 1 [file viruses-13-01325-s001.zip › viruses-1269056-supplementary.pdf]

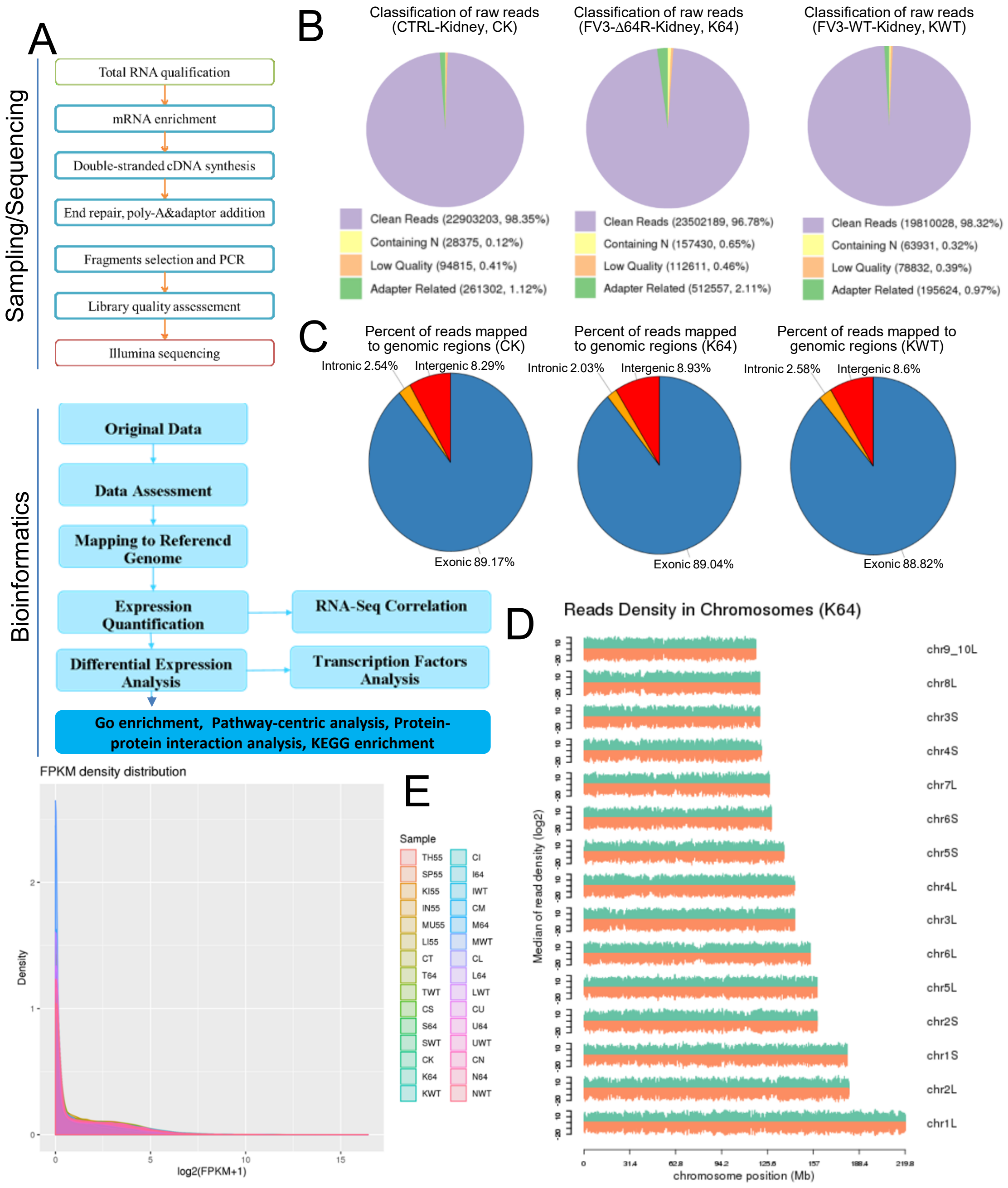

Figure S1. The workflow and data showing general quality and comparability of the RNA-Seq transcriptome data. (A) Workflow of the Illumina RNA-Seq procedure and bioinformatic analysis. (B), (C) and (D) The composition and comparability of raw reads (B), percent of reads mapped to genomic regions (C), and reads density and coverage in Chromosome (D) in representative samples. Very similar reads coverage and distribution were comparatively obtained in all tissue samples in general. (E) FPKM density distribution to show different gene expression levels under different experiment conditions. FPKM distribution, the x-axis shows the $\log_{10}$(FPKM+1) and the y-axis shows gene density. FPKM, Fragments Per Kilobase Million for paired-end RNA-Seq. Please refer the determination for sample types of abbreviations in the text and figure legends as indicated.

Table S1. Software List for data bioinformatic analysis.

Analysis	Software	Version	Parameters	Remarks
Mapping	HISAT2	2.1.0-beta	mismatch = 2	mapping to a reference
Quantification	HTSeq	v0.6.1	-m union	
Differential Expression Analysis	DEGSeq	1.12.0	$\vert \log_2 foldchang \vert >1$ && qvalue<0.005	For sample with bio-replicate using DESeq, samples without bio-replicate using DEGSeq. EdgeR for specific conditions.
	DESeq	1.10.1	padj<0.05	
	edgeR	3.0.8	padj<0.05	
GO Enrichment	GOSeq, topGO,hmmscan	Release2.12	Corrected P-Value<0.05	hmmscan
KEGG Enrichment	KOBAS	v3.0	Corrected P-Value<0.05	
Protein-Protein Interaction Analysis	BLAST	v2.2.28	e-value = 1e-10 && string score >700	Using blast, String database
